# SPECT/CT peak tracer uptake is a strong predictor of aseptic loosening in painful total knee arthroplasty

**DOI:** 10.1007/s00259-026-07775-9

**Published:** 2026-01-30

**Authors:** Helmut Rasch, Randa Elsheikh, George M Avram, Alexandra Leica, Sabrina Chelli, Felix Amsler, Andrej M Nowakowski, Rolf Huegli, Michael T. Hirschmann

**Affiliations:** 1 Institute of Radiology and Nuclear Medicine, Kantonsspital Baselland, Bruderholz, CH-4101 Switzerland; 2 Present Address: University Department of Orthopedic Surgery and Traumatology, Kantonsspital Baselland, Bruderholz, CH-4101 Switzerland; 3 Present Address: Department of Clinical Research, Research Group Michael T. Hirschmann, Regenerative Medicine & Biomechanics, University of Basel, Basel, CH-4001 Switzerland; 4 Amsler Consulting, Basel, Switzerland

**Keywords:** SPECT/CT, Femur peak, Tibia peak, Tracer uptake, Aseptic loosening, Knee arthroplasty

## Abstract

**Purpose:**

This study aims to evaluate the diagnostic accuracy of quantitative three-dimensional Single-Photon Emission Computed Tomography (SPECT/CT) analysis for detecting aseptic loosening after total knee arthroplasty (TKA) and to establish component-specific quantitative thresholds.

**Methods:**

In this retrospective cohort study, patients with painful TKA who underwent standardized assessment, including SPECT/CT after the exclusion of infection, were analyzed. Imaging was performed on a Symbia Intevo Bold (Siemens) and reconstructed using the xSPECT Quant algorithm. Component-specific volumetric uptake parameters, including Maximum, Peak, Mean, and Metabolic Volume, were quantified. Stepwise logistic regression was applied to identify the parameter with the highest diagnostic performance. Optimal cutoff values were established using the Youden Index and the Closest Top method.

**Results:**

Of 107 screened patients, 86 were included in the final analysis. Intraoperative findings confirmed component loosening in 25 cases. Peak uptake showed the highest diagnostic performance for both femoral and tibial components. For femoral loosening, a Peak uptake threshold of 77.2 kBq/ml provided the most balanced diagnostic performance, yielding a sensitivity of 70.6%, specificity of 66.7%, and a negative predictive value (NPV) of 90.2%. For tibial loosening, a threshold of 89.5 kBq/ml achieved a sensitivity of 72.7%, specificity of 80%, and an NPV of 95.2%. The area under the curve was 0.87 for tibial loosening and 0.80 for femoral loosening.

**Conclusion:**

Quantitative SPECT/CT using Peak uptake analysis enables objective and reproducible diagnosis of aseptic loosening. The identified component-specific thresholds show strong predictive value, though the diagnostic accuracy was lower for the femoral component, emphasizing the need for individualized assessment and further prospective validation.

## Introduction

Persistent pain following total knee arthroplasty (TKA) is an increasing clinical and economic challenge. National projections estimate a 43% increase in the demand for primary TKA by 2050, with revision procedures expected to increase by 90% [1]. Despite generally favorable outcomes, up to 10% of patients remain dissatisfied after surgery [2]. Clinically, prosthesis failure is often multifactorial, with aseptic component loosening being among the most common causes [3]. Long-term data indicate that approximately 13% of primary TKA require revision within ten years [4].

Given this multifactorial etiology, a standardized diagnostic algorithm addressing all potential causes is essential [3, 5–9]. Over the past 15 years, Single-Photon Emission Computed Tomography/Computed Tomography (SPECT/CT) has played an increasingly important role in the workup of painful TKAs [10–14]. Combining functional information from SPECT with the anatomical detail from CT allows precise localization of abnormal uptake in relation to specific prosthetic structures.

However, diagnosing prosthetic loosening using SPECT/CT remains challenging. Increased uptake can be seen not only in aseptic loosening, but also in infection and malalignment [13, 15]. Importantly, as previously shown [16], uptake patterns can persist for many years, further complicating visual assessment. Previous studies have sought to identify specific uptake patterns correlating with aseptic loosening, infection, or other causes of pain, sometimes incorporating biomechanical axis information from CT [3, 17, 18].

Historically, SPECT uptake has been assessed semiquantitatively, using color scales or relative to a reference region [5, 19]. While informative, these methods are limited by poor reproducibility, inter-observer variability, and methodological complexity. Absolute quantification could overcome these limitations, but barriers such as complex calibration, limited software availability, and sparse clinical evidence have hindered widespread adoption [20]. Since its introduction in 2013, quantitative SPECT/CT with xSPECT Quant (Siemens) has simplified measurements, allowing uptake to be reported in standardized uptake values (SUV) or absolute units (kBq/ml) [21].

In this context, the present study aims to evaluate whether prosthetic component loosening can be reliably diagnosed using quantitative SPECT/CT uptake measurements based on volumes of interest (VOI) around prosthesis components and assess which uptake parameter is most predictive of loosening. As a secondary objective, the study seeks to establish potential uptake cutoff values that may support diagnostic decision-making, improve comparability, and reduce investigator-dependent variability.

## Methods

### Study design

The present study follows the diagnostic test accuracy methodology outlined in the Standards for Reporting of Diagnostic Accuracy (STARD) guidelines for transparent reporting [22]. Ethical approval was granted by the EKNZ Committee under the registration number 2018–02020. The study was performed in accordance with the principles of the 1964 Declaration of Helsinki and its later amendments and in accordance with the Good Clinical Practice Guidelines.

Patients were retrospectively identified from the authors’ hospital registry and checked against the following inclusion criteria: (1) painful TKA, (2) approved and signed informed consent, and (3) complete diagnostic work-up for revision TKA consisting of anteroposterior, lateral, and stress radiographs, and SPECT/CT with 3D alignment assessment [5, 23–25]. Exclusion criteria included revision surgeries performed for infection, soft-tissue trauma, periprosthetic fracture, implant failure through polyethylene wear, or inlay change. Potential confounders, including the interval from initial operation to symptom onset or suspected loosening, and concomitant conditions such as tibiofemoral instability, patella baja, retropatellar arthrosis, and arthrofibrosis, were recorded and systematically incorporated as covariates in the analysis of tracer uptake.

### SPECT/CT protocol

All patients underwent a standardized triphasic bone scintigraphy following intravenous administration of 700 MBq of 99 m-Tc-HDP (Oxidronate, Curium). The imaging protocol comprised a planar perfusion phase, a blood-pool phase with SPECT acquisition, and a delayed phase with whole-body planar imaging combined with SPECT/CT of both knees. Data acquisition was performed on a SPECT/CT system (INTEVO Bold, Siemens). SPECT was acquired with a 256 × 256 matrix, 60 angular steps, and 12 s per frame. Image reconstruction was carried out using 3D OSEM Flash, and, for quantification, xSPECT Quant was applied. To ensure quantitative accuracy, system calibration was verified monthly using a 57Co point source (​nominal activity certified by the National Institute of ​Standards and Technology, Gaithersburg, MD), following the ​manufacturer’s specifications for energy and sensitivity [21].

### Image evaluation and quantification

**Fig. 1:**
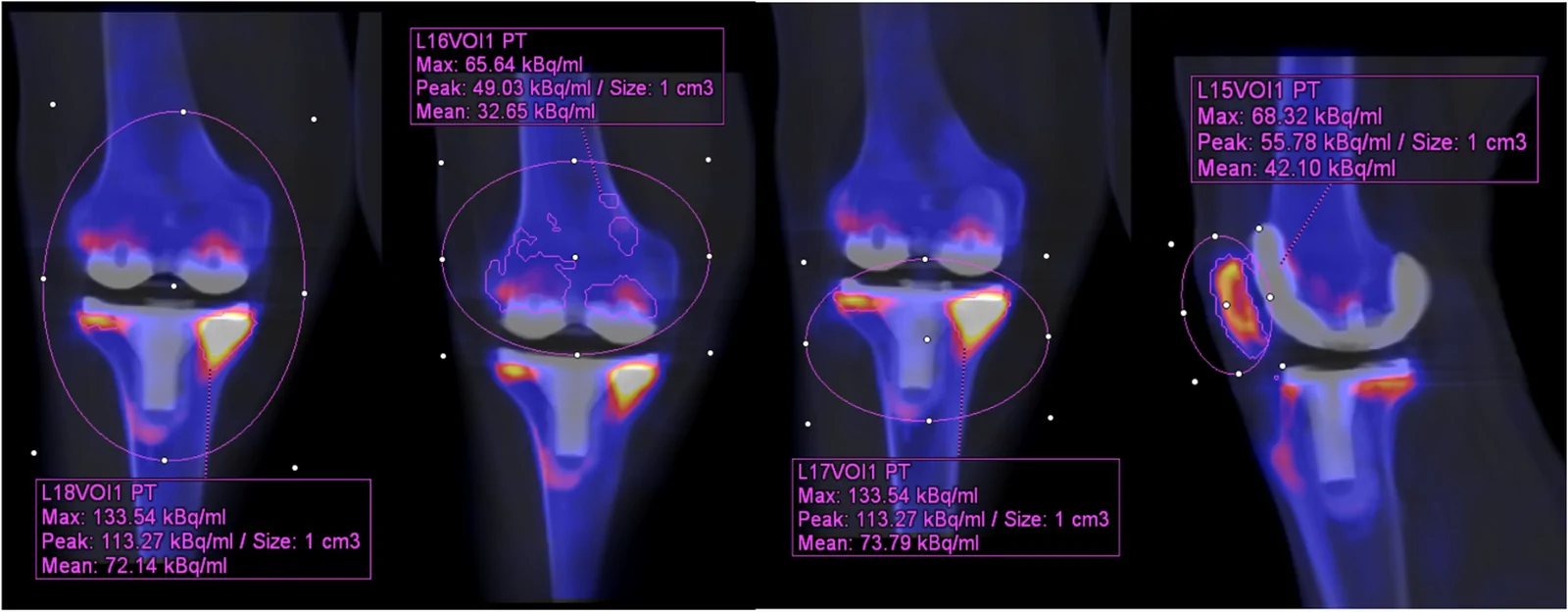
VOI delineation for SPECT/CT analysis, illustrating component-specific VOIs around the femoral and tibial components, the patella, and a composite VOI covering the entire prosthesis

Evaluation of SPECT data was performed using a SyngoVia workstation (Version VB60, Siemens). For quantitative analysis, the VOI isocontour tool was applied. VOIs were delineated around the femoral and tibial components, the patella, and the entire prosthesis (Fig. 1). For each VOI, the following quantitative uptake parameters (kBq/ml) were recorded: Maximum Uptake (Max), Peak (highest mean within a 1cm^3^ volume), Mean, and Metabolic Volume (MTV). VOIs were generated using an automated autocontouring algorithm, and boundaries were standardized by applying a threshold of 50% of the maximum uptake. In cases where automatic delineation was insufficient to accurately capture the structure or adjacent hypermetabolic areas, manual adjustments were conducted.

All measurements were performed by a single reader and independently cross-validated by a nuclear medicine physician with over 15 years of experience in SPECT/CT interpretation.

### Diagnostic reference standard

In all included patients, aseptic loosening was suspected based on clinical evaluation using the Bruderholz diagnostic algorithm [5, 19, 24], complemented with SPECT/CT findings. The definitive diagnosis of loosening was established intraoperatively and served as the diagnostic gold standard. Loosening was confirmed by direct visualization of fluid motion during manipulation, or lack of stable fixation at the bone-implant interface [26]. All revision procedures were performed by a single senior orthopedic surgeon (MTH) to ensure consistency of intraoperative assessment.

### Statistical analysis

For each measured parameter (Max, Peak, Mean activity concentration, and MTV), values were summarized as mean ± standard deviation. To evaluate the interdependence among the measured parameters and detect potential multicollinearity, Pearson’s correlation analyses were performed. Potential confounders identified preoperatively were evaluated for correlations with the main quantitative parameters. Comparisons of mean values between positive and negative loosening cases were performed using two-tailed independent-samples t-tests. For each parameter, adjusted R2 values and effect sizes (Cohen’s d) were calculated to quantify association strength and identify the most relevant predictor of aseptic loosening.

Receiver operating characteristic (ROC) analyses were performed to evaluate the discriminative ability of each parameter for femoral and tibial loosening, and the area under the curve (AUC) with 95% confidence intervals (CI) was calculated. The Youden index and the closest top method were applied to identify the optimal cutoff for each uptake parameter, and diagnostic performance was assessed by calculating sensitivity, specificity, positive predictive value (PPV), negative predictive value (NPV), and likelihood ratios. Stepwise logistic regression was conducted to estimate odds ratios (OR) with 95% CIs for the most relevant predictor variables identified in the preliminary analyses. Statistical significance was set at *p* < 0.05. All analyses were performed using SPSS (IBM SPSS Statistics for Windows, Version 26.0, Armonk, NY: IBM Corp).

## Results

### Patient demographics

**Fig. 2:**
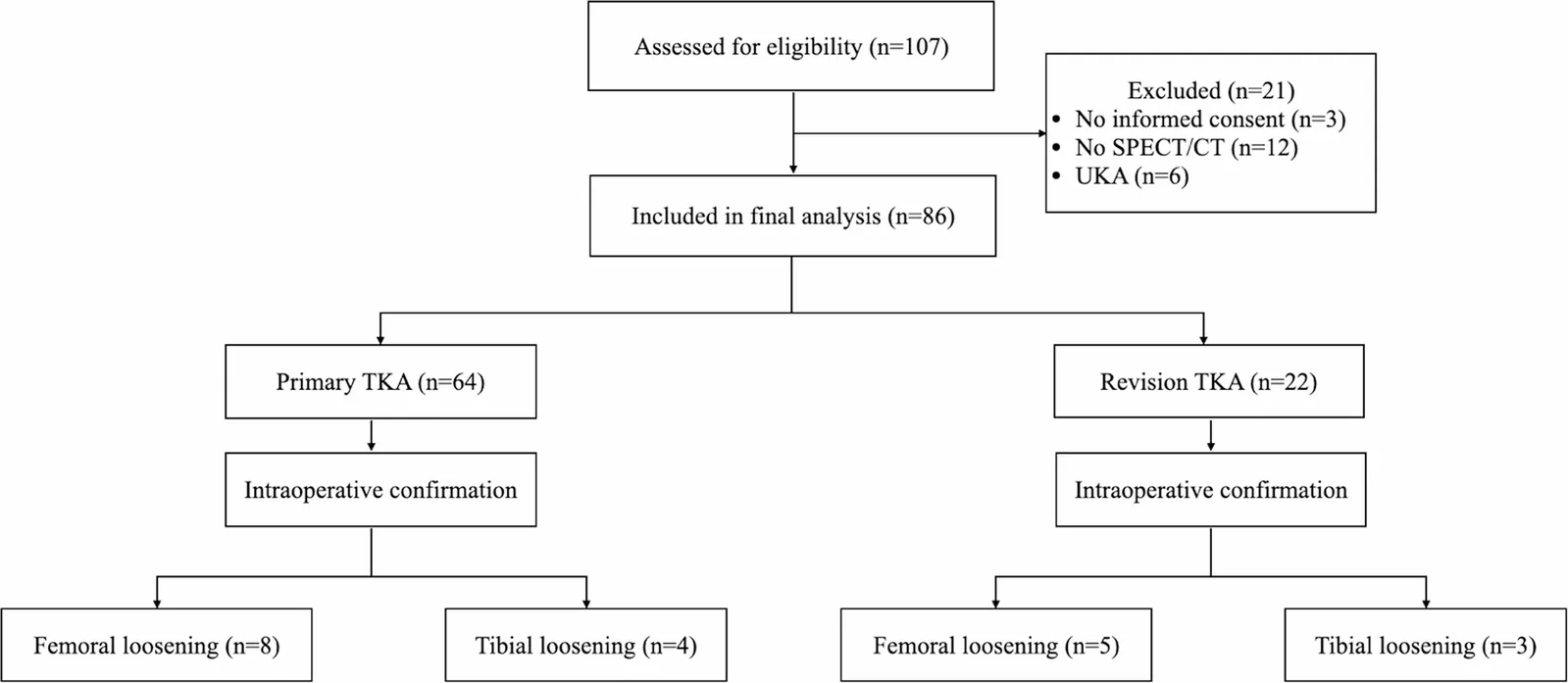
Flow diagram illustrating patients’ enrollment process

A total of 86 patients were included in the final analysis (Fig. 2). Most procedures were primary TKAs (*n* = 64, 74.4%), and 22 (25.6%) were revision cases. The mean age was 67.6 ± 9.4 years; 35 patients were males (40.7%) and 51 females (59.3%), with no significant demographic differences between groups (*p* > 0.44).

Component loosening was identified intraoperatively in 25 patients (29.1%). Femoral loosening was present in 17 (19.8%) cases, tibial loosening in 11 (12.8%), and patellar loosening in 2 (2.3%). Combined femoral and tibial loosening occurred in 4 patients (4.7%). The prevalence of component loosening did not differ significantly between primary and revision cases (*p* > 0.38).

### Diagnostic performance of quantitative SPECT parameters

Quantitative SPECT uptake parameters (Max, Peak, Mean, and MTV) were measured independently for each prosthetic component and the entire prosthesis. Interdependence analysis revealed strong correlations among Max, Peak, and Mean values for femur, tibia, and patella (Pearson *r* = 0.92–1.00), whereas MTV showed weak inverse correlations (*r* = − 0.25 to − 0.37). Cross-component correlations were moderate (*r* = 0.25–0.48), suggesting that metabolic activity patterns were largely component-specific.

**Fig. 3:**
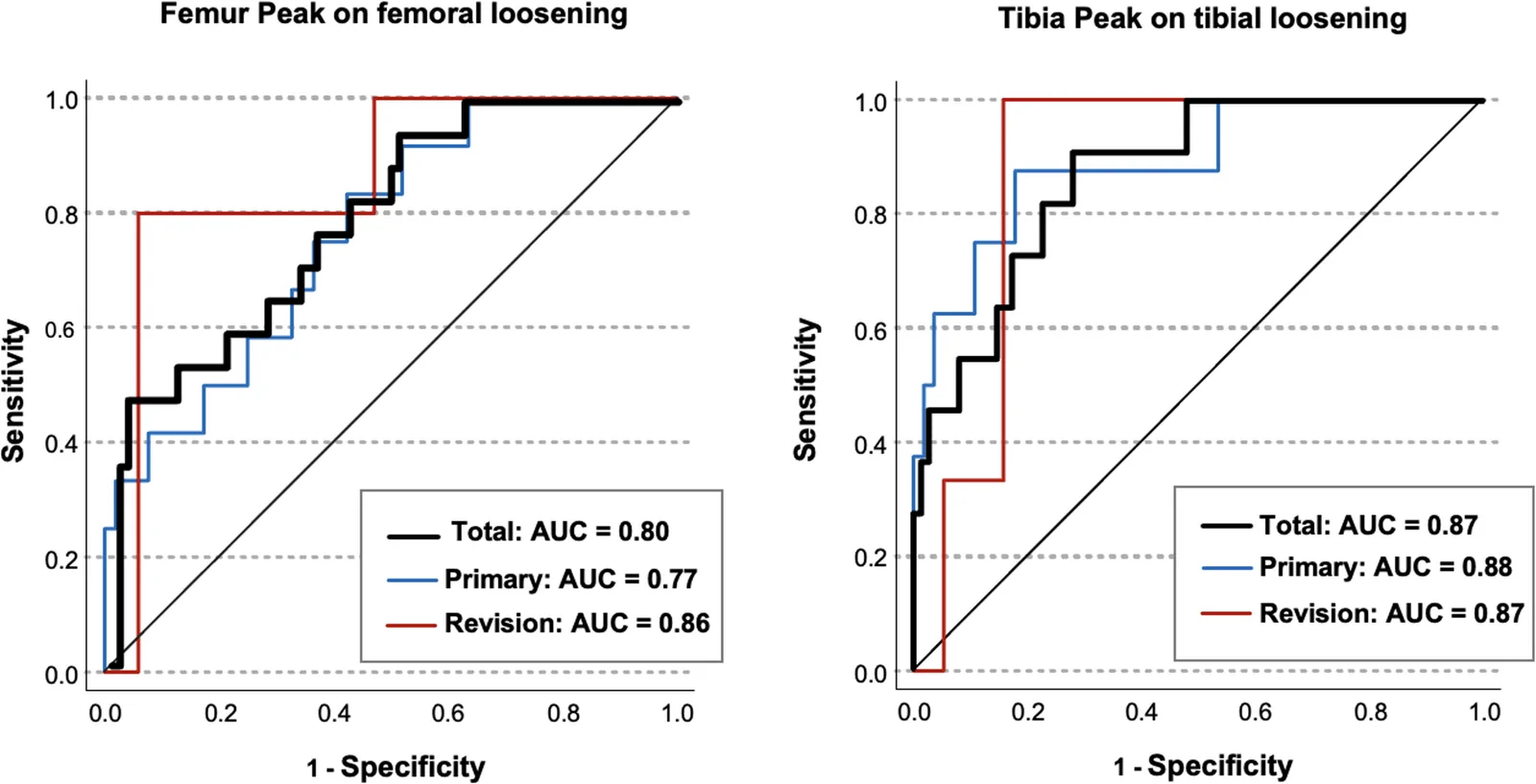
ROC curve illustrating the diagnostic performance of Femur Peak (left) and Tibia Peak (right) (kBq/ml/cm^3^)

Peak uptake exhibited the strongest discrimination between loosening and non-loosening. Femoral loosening showed substantially higher Peak values than stable components (102.5 ± 37 vs. 67.7 ± 31.1 kBq/ml; Cohen’s *d =* 1.02). Similarly, tibial loosening was associated with markedly elevated Peak values (107.7 ± 28.8 vs. 67 ± 23.5 kBq/ml; Cohen’s *d =* 1.55) (Table 1). ROC analysis further confirmed the discriminative ability of Peak uptake, with an AUC of 0.80 (95% CI 0.68–0.91) for femoral and 0.87 (95% CI 0.77–0.96) for tibial components (Fig. 3).

**Table 1:** Bone Tracer Uptake of confirmed and unconfirmed cases of femoral and tibial aseptic loosening

Femoral loosening	No (*N*=69)	Yes (*N*=19)		
	Mean (SD)	Mean (SD)	R^2^	Cohen's d
Femur Peak - kBq/ml / cm^3^	67.7 (31.1)	102.5 (37)	0.16	1.02
Femur Max - kBq/ml	89.3 (39.6)	125.1 (43.6)	0.11	0.89
Femur Mean - kBq/ml	55 (25.9)	78.3 (26.8)	0.11	0.86
Femur Min - kBq/ml	44.7 (20)	62.6 (21.9)	0.11	0.86
Femur Swelling - kBq/ml 50%	44.7 (19.9)	62.6 (21.9)	0.11	0.86
Femur Volume (MTV) cm^3^	16.9 (15.9)	28.7 (15)	0.08	0.76
Femur SD - kBq/ml	9.2 (5)	12.9 (5.1)	0.08	0.75
Tibial loosening	No (*N*=75)	Yes (*N*=11)		
	Mean (SD)	Mean (SD)	R^2^	Cohen's d
Tibia Peak - kBq/ml/ cm^3^	66.9 (23.5)	107.7 (28.8)	0.25	1.55
Tibia Mean - kBq/ml	55.3 (19.7)	83.7 (18.9)	0.19	1.47
Tibia Swelling - kBq/ml 50%	44.4 (15.5)	66.1 (14.7)	0.18	1.43
Tibia Max - kBq/ml	88.8 (31.1)	132.2 (29.7)	0.18	1.43
Tibia Min - kBq/ml	44.5 (15.7)	66.1 (14.8)	0.18	1.42
Tibia SD - kBq/ml	9.2 (3.6)	14.4 (3.9)	0.19	1.40
Tibia Volume (MTV) cm^3^	20.3 (13.1)	19.2 (8.8)	0.00	0.10

### Overall diagnostic accuracy of peak values

Stepwise logistic regression identified Femur Peak (OR = 1.03, 95% CI 1.01–1.05, *p* < 0.01) and Tibia Peak (OR = 1.07, 95% CI 1.03–1.10, *p* < 0.01) as best predictors of component loosening (Table 2).

When applying thresholds across the entire cohort, the Youden Index and the Closest Top method yielded distinct diagnostic profiles. For Femur Peak, the Closest Top cutoff of 77.2 kBq/ml produced a sensitivity of 70.6% and specificity of 66.7%, with an NPV of 90.2% and a PPV of 34.3%. The Youden threshold of 100.9 kBq/ml increased specificity to 97.1% but reduced sensitivity to 47.1%, with a PPV of 80% and NPV of 88.2%.

For Tibia Peak, the Closest Top cutoff of 89.5 kBq/ml achieved a sensitivity of 72.7% and specificity of 80% (NPV 95.2%, PPV 34.8%). The Youden cutoff of 77.9 kBq/ml improved sensitivity to 90.9%, with specificity of 70.7% (NPV 98.1%, PPV of 31.3%).

**Fig. 4:**
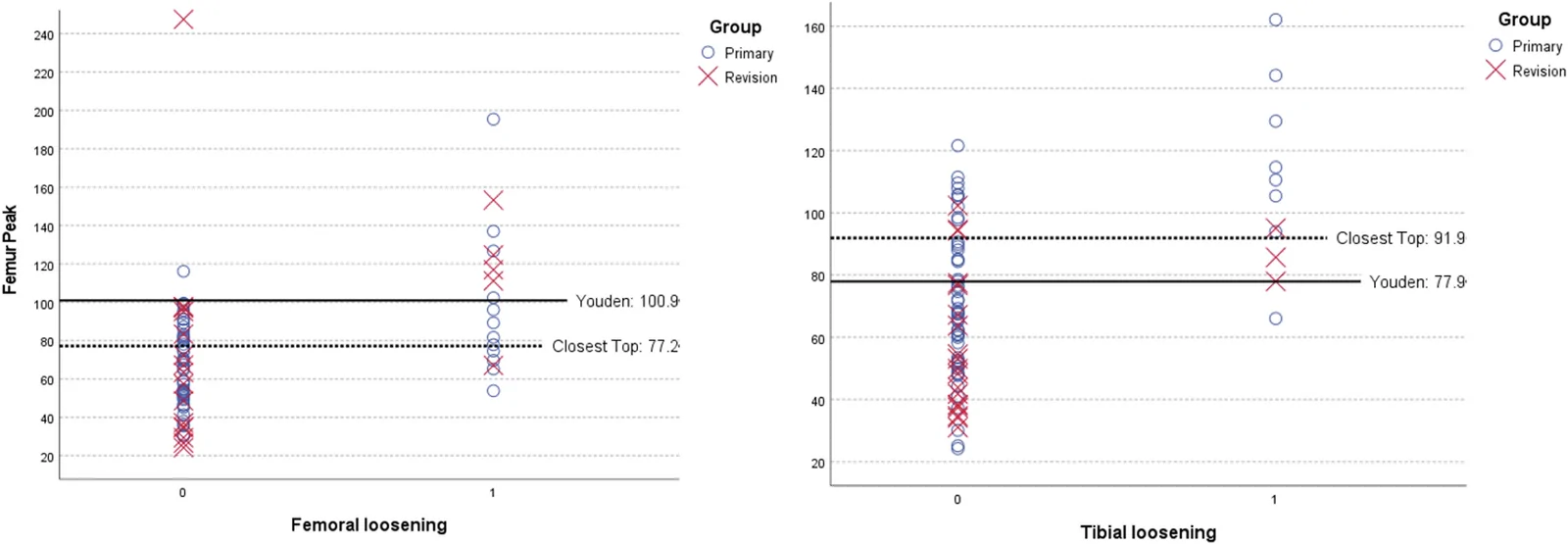
Optimal Femur and Tibia Peak thresholds (kBq/ml/cm^3^) for patients with loosened primary or revised total prostheses

Stratified analysis revealed that optimal metabolic thresholds for primary and revision implants were comparable to the overall cutoffs. Femoral component thresholds ranged from 74.5 to 104.4 kBq/ml, while tibial component thresholds ranged from 77.7 to 92.3 kBq/ml (Fig. 4).

### Effect of concomitant conditions on uptake values

Pearson correlation analysis revealed that potential confounders, including tibiofemoral instability, patella baja, retropatellar arthrosis, and arthrofibrosis, had no influence on uptake values, except for a weak negative correlation of instability with femoral peak (*r* = − 0.22) and tibia volume (*r* = − 0.25). Similarly, the interval from surgery to symptom onset was not significantly correlated with femoral (*r* = − 0.007, *p* = 0.958) or tibial (*r* = − 0.031, *p* = 0.822) uptake.

## Discussion

The key finding of the present study is that Peak uptake emerged as the most discriminative quantitative SPECT/CT parameter for detecting aseptic loosening in TKA, demonstrating high diagnostic accuracy for both the femoral (AUC 0.80) and the tibial (AUC 0.87) components. Peak uptake outperformed other quantitative markers, likely owing to its lower susceptibility to image noise and independence from arbitrary thresholding, which enhances reproducibility and clinical applicability [27]. Distinct optimal thresholds were identified for the femoral and tibial components, largely overlapping between primary and revision arthroplasties, underscoring the need for component-specific evaluation.

A femoral Peak cutoff of 77.2 kBq/ml provided a balanced diagnostic profile, with a sensitivity of 70.6% and specificity of 66.7%, while a tibial threshold of 89.5 kBq/ml achieved a more favorable balance, yielding a sensitivity of 72.7% and specificity of 80%. The comparatively lower sensitivity observed for femoral components likely reflects residual metal-induced artifacts along the bone-implant interface, which remain incompletely corrected despite the use of iterative metal artifact reduction (iMAR) algorithms [28]. Such artifacts can cause localized photon starvation and beam-hardening effects, resulting in local signal attenuation and underestimation of true tracer activity [29]. Although prior work suggests that iMAR introduces minimal quantitative distortion in PET-derived activity measurements [30–32], persistent streak artifacts in the anterior and posterior femoral shield regions remain a limitation. Careful VOI placement, with avoidance of artifact-prone zones, mitigates these effects, and the high NPV observed in our study supports the method’s utility as a rule-out metric for femoral loosening, even in the presence of residual metal artifacts.

Our findings demonstrate that volumetric VOI-based quantification enables objective and reproducible assessment of tracer uptake, reducing observer dependence inherent to visual or semi-quantitative analyses. This supports a growing body of literature advocating quantitative SPECT/CT as an advanced diagnostic tool for prosthetic loosening. Al-Nabhani et al. analyzed 105 unicompartmental and total knee arthroplasties and reported sensitivity and specificity of 100% and 95%, respectively. Yet, only a subset of patients underwent intraoperative verification, limiting the robustness of their findings [33]. A more methodologically comparable approach was proposed by Braun et al., who employed a VOI-based quantification technique encompassing the entire prosthesis. Their study yielded a sensitivity of 90%, specificity of 71.4%, and NPV of 95%, closely mirroring our results. Nevertheless, their mixed cohort of hip and knee prostheses, lack of component-level differentiation, and limited intraoperative confirmation hinder generalizability [34]. By contrast, our component-specific analysis reveals that femoral, tibial, and patellar uptake values showed only weak intercorrelation, suggesting that loosening is a localized process requiring independent evaluation of each component. This observation may explain discrepancies among previous studies that analyzed the prosthesis as a single unit, masking component-specific thresholds and patterns.

When interpreting increased tracer uptake, the potential influence of concomitant mechanical or inflammatory conditions must be considered. Instability, patellar maltracking, arthrofibrosis, and anterior compartment overload may elevate uptake in the absence of loosening. In our cohort, instability showed a weak inverse correlation with femoral uptake, whereas other conditions had no significant association. Correlation with the CT component remains essential to distinguish these entities, reinforcing one of the key advantages of hybrid SPECT/CT over planar scintigraphy [34].

Several limitations should be acknowledged. Subgroup analyses by fixation method were not feasible due to limited sample size, although data from hip arthroplasty suggest that fixation type may influence periprosthetic uptake, particularly in the early postoperative period [35, 36]. Similarly, although separate cutoffs could be identified for primary and revision prostheses, the limited number of loosening in each subgroup limits the reliability of these values and warrants a careful interpretation. Large, prospective studies are needed to validate such thresholds and confirm the absence of major procedure-based uptake differences. Moreover, the SyngoVia (VB60) software restricts VOI definition to ellipsoidal geometries, limiting precise delineation of the bone-cement or bone-implant interface. Implementation of freehand VOI tools could further enhance quantitative precision. In addition, incidental inclusion of hypermetabolic structures, such as ossifications, tendinopathies, or overloaded cortical areas within the VOI, could have resulted in overestimated uptake values in isolated cases. Finally, internal validation of the diagnostic models using bootstrap resampling was not performed, which limits the precision and confidence of the reported AUC estimates. Future studies with larger cohorts should incorporate resampling or external validation to strengthen the robustness and generalizability of the findings.

Despite these limitations, this study provides strong evidence that quantitative SPECT/CT with Peak uptake analysis offers an accurate, reproducible, and clinically applicable method for diagnosing or excluding prosthetic loosening after TKA. Incorporating this technique into diagnostic workflows could improve diagnostic confidence, reduce unnecessary revision surgeries, and establish quantitative benchmarks for multicenter and longitudinal studies.

## Conclusion

Quantitative SPECT/CT with VOI-based Peak uptake analysis enables objective and reproducible assessment of aseptic loosening after TKA. Peak uptake offers the highest diagnostic discrimination for both femoral and tibial components, supporting the need for component-specific interpretation. While demonstrating the clinical importance of quantitative SPECT/CT, the proposed thresholds should be regarded as exploratory and validated in larger multicenter cohorts before being adopted into routine diagnostic algorithms.

**Table 2:** Stepwise logistic regression and diagnostic accuracy metrics for femoral and tibial loosening

Variables	B	*p*	Odds ratio Exp (β)	95% CI	Chi^2^	*p*	Nagelkerke R^2^
Femur Peak	0.029	0.004	1.03	1.01–1.05	12.4	< 0.001	0.21
Constant	-3.755	0.000	0.02				
Tibia Peak	0.063	0.001	1.07	1.03–1.10	21.1	< 0.001	0.41
Constant	-7.395	< 0.001	0				

## Data Availability

The dataset analyzed during the current study will be made available from the corresponding author upon reasonable request and with approval from the ethics committee.

## Abbreviations

SPECT/CT: Single Photon Emission Computed Tomography/Computed Tomography
TKA: Total Knee Arthroplasty
SUV: Standardized Uptake Values
VOI: Volume of Interest
STARD: Standards for Reporting of Diagnostic Accuracy
ROC: Receiver Operating Curve
AUC: Area Under the Curve
PPV: Positive Predictive Value
NPV: Negative Predictive Value
MTV: Metabolic Volume
iMAR: iterative Metal Artifact Reduction
